# Efficacy and safety of bevacizumab in elderly patients with metastatic colorectal cancer: results from the Czech population-based registry

**DOI:** 10.1186/1471-230X-14-53

**Published:** 2014-03-25

**Authors:** Lubomir Slavicek, Tomas Pavlik, Jiri Tomasek, Zbynek Bortlicek, Tomas Buchler, Bohuslav Melichar, Rostislav Vyzula, Jana Prausova, Jindrich Finek, Ondrej Majek, Ladislav Dusek

**Affiliations:** 1 Department of Oncology, Hospital Jihlava, Jihlava, Czech Republic; 2 Institute of Biostatistics and Analyses, Masaryk University, Brno, Czech Republic; 3 Department of Comprehensive Cancer Care, Masaryk Memorial Cancer Institute, Brno, Czech Republic; 4 Department of Oncology and First Faculty of Medicine, Charles University and Thomayer Hospital, Prague, Czech Republic; 5 Department of Oncology, Palacky University Medical School and Teaching Hospital, Olomouc, Czech Republic; 6 Department of Oncology, Motol Hospital and Charles University, Prague, Czech Republic; 7 Department of Oncology, University Hospital Pilsen, Pilsen, Czech Republic

**Keywords:** Anti-angiogenic therapy, Chemotherapy, Elderly patients, Overall survival, Progression-free survival

## Abstract

**Background:**

Patients aged 65 years and older represent the majority of patients with metastatic colorectal cancer (mCRC). However, this patient population is often underrepresented in clinical trials and probably undertreated in the clinical practice.

**Methods:**

We have analysed the outcomes of 3,187 mCRC patients treated with first-line bevacizumab based on data from the Czech national registry of mCRC patients aiming to compare the treatment efficacy and safety according to the age categories.

**Results:**

In total, 2,126 (66.7%), 932 (29.2%), and 129 (4.0%) patients were aged <65 years, 65 to 75 years, and 75+ years, respectively. Median progression-free survival (PFS) was 11.4, 11.3, and 11.8 months for patients aged <65 years, 65 to 75 years, and 75+ years, respectively (p = 0.94). Median overall survival (OS) was 26.9, 27.5, and 25.1 months for patients aged <65 years, 65 to 75 years, and 75+ years, respectively (p = 0.73). Using multivariable Cox model for both PFS and OS, the patient age was not significantly associated with either PFS or OS. No increase in bevacizumab-related toxicity was observed among the elderly mCRC patients with the exception of hypertension, which was observed in 71 (3.3%), 34 (3.6%), and 10 (7.8%) patients aged <65 years, 65 to 75 years, and 75+ years, respectively.

**Conclusions:**

The results of the present study suggest similar outcome in terms of OS and PFS with bevacizumab-containing therapy in elderly mCRC patients fit for chemotherapy combined with targeted therapy compared to younger patients. Thus, chronological age should not be considered to represent a limitation in prescribing bevacizumab-containing therapy in mCRC patients.

## Background

Colorectal cancer (CRC) represents a serious public health problem in the Czech Republic as the Czech population presently ranks 3^rd^ in international statistics of age-standardised CRC incidence rates, with 78 new cases of CRC being diagnosed annually per 100,000 inhabitants (2010) [1]. In addition, more than one quarter of these patients have metastatic disease at the time of diagnosis [2]. Over the past decade, however, the introduction of new cytotoxic drugs, targeted therapy, and an increase in the use of liver resection have resulted in significantly improved outcomes in metastatic CRC (mCRC) patients [3,4]. Monoclonal antibodies, the targeted agents currently used in the treatment of mCRC are usually utilised in combination with cytotoxic drugs. The first and currently most widely used monoclonal antibody in mCRC therapy is bevacizumab (F. Hoffman-La Roche Ltd., Basel, Switzerland), a drug targeting the vascular endothelial growth factor. Efficacy and safety of bevacizumab administered in combination with chemotherapy backbone regimens in patients with mCRC have been the subject of several randomised clinical trials [5-7] as well as observational studies [8,9]. Although patients ≥65 years of age represent the majority of patients with mCRC, this patient population is often underrepresented in clinical trials and very likely undertreated in the clinical practice [10,11]. However, the results of recently published randomised trials as well as observational studies [12-15] suggest that bevacizumab provides similar overall survival (OS) and progression-free survival (PFS) benefits in patients aged ≥65 years compared to younger patients.

In the present study, we have analysed the data from the Czech national registry of mCRC patients treated with first-line bevacizumab with the aim to compare the treatment outcomes according to age.

## Methods

### Patients

Adult mCRC patients treated with first-line bevacizumab-containing therapy in the Czech Republic were included in the present analysis. In the Czech Republic, the administration of targeted therapy is concentrated to comprehensive cancer centres and these drugs are reimbursed only when administered in one of these centres. The data set was obtained from the Czech population-based, retrospective, observational CORECT registry [16] which contains de-identified data of the Czech mCRC patients treated with targeted therapies including bevacizumab, cetuximab, and panitumumab. The protocol was approved by the independent ethics committee at each participating centre (Ethics Committee (EC) of the Ceske Budejovice Hospital, EC of the Chomutov Hospital, EC of the General University Hospital in Prague, EC of the Jihlava Hospital, EC of the Liberec Regional Hospital, EC of the Masaryk Hospital in Usti nad Labem, EC of the Masaryk Memorial Cancer Institute in Brno, EC of the Na Bulovce Hospital in Prague, EC of the Na Homolce Hospital in Prague, EC of the Novy Jicin Hospital, EC of the Pardubice Regional Hospital, EC of the St. Anne’s University Hospital (UH) in Brno, EC of the Thomayer Hospital in Prague, EC of the Tomas Bata Regional Hospital in Zlin, EC of the UH Brno, EC of the UH Hradec Kralove, EC of the UH in Motol, Prague, EC of the UH Olomouc, EC of the UH Ostrava, EC of the UH Pilsen) and complied with the International Ethical Guidelines for Biomedical Research Involving Human Subjects, Good Clinical Practice guidelines, the Declaration of Helsinki, and local laws. Based on the recent validation using the data of all health care payers in the Czech Republic, the CORECT database includes data of approximately 96% of all mCRC patients treated with targeted therapies in the country. The data are entered into the CORECT database by the clinicians and updated at least twice yearly. The final data cut-off date was 30 September 2012.

### Outcome assessment

Both OS and PFS were considered the primary efficacy measures in the present study. Objective response was assessed using the RECIST criteria. Both OS and PFS were calculated from the start of bevacizumab-containing therapy. Only patients who started bevacizumab and chemotherapy at least six months prior to the data cut-off were included in the present analysis. Such design ensured sufficient follow-up for statistically relevant analyses of the time-to-event endpoints.

Adverse events were assessed using the Common Terminology Criteria for Adverse Events (CTCAE) version 3.0 criteria. The severity of adverse events was classified by the attending medical oncologist as ‘mild to moderate’ corresponding to grade 1 to 2 toxicity or ‘severe’ corresponding to grade 3 to 4 toxicity. Only toxicities considered to be related to the administration of bevacizumab therapy were entered into the database.

In accordance with the World Health Organization, elderly mCRC patients were defined as persons aged ≥65 years. In some studies, however, the cut-off to define elderly population was set at age of 75 years. Therefore, in the present study outcomes were analysed based on the patients’age at the start of bevacizumab therapy in following subgroups: (1) <65 years, (2) 65 to 75 years, and (3) ≥75 years.

### Statistical analysis

Standard descriptive statistics were used to characterise the data. Differences in categorical parameters as well as in the incidence of adverse effects among age categories were assessed using the Pearson chi-square test. Comparisons of continuous variables were based on the Kruskal–Wallis test. The survival was estimated using the Kaplan–Meier method. Log-rank test was used to compare OS and PFS for different subgroups. Multivariable Cox proportional hazards model was used to assess the effect of age on survival in the presence of other potential predictive and prognostic factors. Standard level of significance α = 0.05 was used.

## Results

In total, 3,187 mCRC patients treated with first-line bevacizumab were analysed. Of those, 2,126 (66.7%), 932 (29.2%), and 129 (4.0%) patients were age <65 years, 65 to 75 years, and ≥75 years, respectively. Baseline patient characteristics are shown in Table 1. As of 30 September 2012, the median follow-up was 17 months (range 0.5-84.6 months) with 209 (9.8%), 110 (11.8%), and 17 (13.2%) patients aged <65 years, 65 to 75 years, and ≥75 years remaining on bevacizumab-containing therapy, respectively. Median duration of bevacizumab therapy was 7.4 months (range 0.5-58.7 months), 6.9 months (range 0.5-41.7 months), and 6.4 months (range 0.5-31.0 months) in the <65 years, 65 to 75 years, and ≥75 years age cohorts, respectively.

**Table 1:** Baseline characteristics of analysed patients

**Characteristic**	**<65 years (n = 2,126)**	**65-75 years (n = 932)**	**≥ 75 years (n = 129)**	**p-value**^**a**^
**Males,** n (%)	1,324 (62.3)	593 (63.6)	79 (61.2)	0.74
**Age at treatment initiation**				
Median (min-max)	57.7 (21.3-64.9)	68.3 (65.0-74.9)	76.9 (75.0-85.2)	-
**Localization,** n (%)				
Colon	1,287 (60.5)	571 (61.3)	84 (65.1)	0.57
Rectum	839 (39.5)	361 (38.7)	45 (34.9)	
**History of thromboembolism,** n (%)	68 (3.2)	55 (5.9)	7 (5.4)	0.002
**History of hypertension,** n (%)	629 (29.6)	494 (53.0)	85 (65.9)	<0.001
**Primary metastatic,** n (%)				
M0	804 (37.8)	389 (41.7)	55 (42.6)	0.09
M1	1,322 (62.2)	543 (58.3)	74 (57.4)	
**Adenocarcinoma,** n (%)	2,060 (96.7)	916 (98.3)	127 (98.4)	0.07
**Prior surgery,** n (%)	1,690 (79.5)	808 (86.7)	117 (90.7)	<0.001
**Prior radiotherapy,** n (%)	437 (20.6)	200 (21.5)	17 (13.2)	0.09
**Adjuvant chemotherapy,** n (%)	639 (30.1)	302 (32.4)	39 (30.2)	0.43
**Site of metastatic disease,** n (%)				
Liver	1,348 (63.4)	594 (63.7)	99 (76.7)	0.009
Lung	504 (23.7)	240 (25.8)	31 (24.0)	0.48
Other	933 (43.9)	390 (41.8)	41 (31.8)	0.02
**Number of metastatic sites,** %				
1/2/>2	55.5/31.2/13.3	58.6/31.8/9.6	62.7/30.2/7.1	0.02
**Chemotherapy regimens,** n (%)				
FOLFOX	903 (42.5)	394 (42.3)	51 (39.5)	<0.001
XELOX	753 (35.4)	313 (33.6)	25 (19.4)	
FOLFIRI	199 (9.4)	74 (7.9)	5 (3.9)	
XELIRI	127 (6.0)	39 (4.2)	1 (0.8)	
Capecitabine	39 (1.8)	44 (4.7)	18 (14.0)	
5-FU/LV	21 (1.0)	21 (2.3)	23 (17.8)	
Other	67 (3.2)	32 (3.4)	3 (2.3)	
Without CT	17 (0.8)	15 (1.6)	3 (2.3)	
**PS at bevacizumab initiation,** n (%)				
0	612 (28.8)	256 (27.5)	41 (31.8)	0.02
1	549 (25.8)	279 (29.9)	39 (30.2)	
2-3	24 (1.1)	15 (1.6)	6 (4.7)	
Not available	941 (44.3)	382 (41.0)	43 (33.3)	
**Treatment duration** (months)				
Median (min- max)	7.4 (0.5-58.7)	6.9 (0.5-41.7)	6.4 (0.5-31.0)	0.04
**Best response,** n (%)				
CR	332 (15.6)	112 (12.0)	8 (6.2)	0.003
PR	675 (31.7)	271 (29.1)	40 (31.0)	
SD	741 (34.9)	381 (40.9)	59 (45.7)	
PD	248 (11.7)	107 (11.5)	12 (9.3)	
Not available	130 (6.1)	61 (6.5)	10 (7.8)	

^a^Kruskal–Wallis test was used for age at treatment initiation and treatment duration, Pearson chi-square test for the rest of variables.

### Chemotherapy regimens

In most patients across all age subgroups (n = 2,439, 76.5%), bevacizumab was administered in combination with oxaliplatin-based chemotherapy backbone regimens including infusional 5-fluorouracil/leucovorin/oxaliplatin (FOLFOX) and capecitabine/oxaliplatin (XELOX) (Table 1). However, while the percentage of mCRC patients receiving FOLFOX was approximately identical in all age groups, a trend toward decreased use of XELOX in patients aged ≥75 years was observed. In addition, chemotherapy regimens containing irinotecan were less frequently used in patients aged ≥75 years. On the other hand, fluoropyrimidine monotherapy was more likely to be used as chemotherapy backbone in patients aged ≥75 years, and 17.8% and 14.0% of these patients received a 5-fluororuracil/leucovorin (5-FU/LV) regimen or capecitabine, respectively.

### Survival outcomes

**Figure 1:**
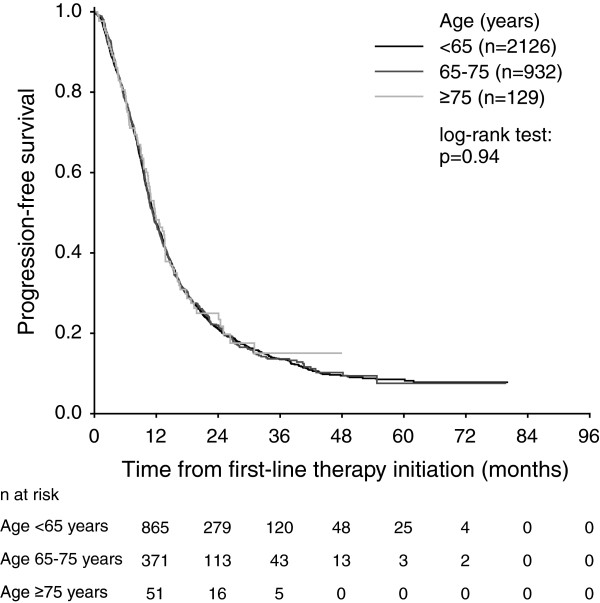
Progression-free survival of metastatic colorectal cancer patients treated with first-line bevacizumab according to the age categories.

**Figure 2:**
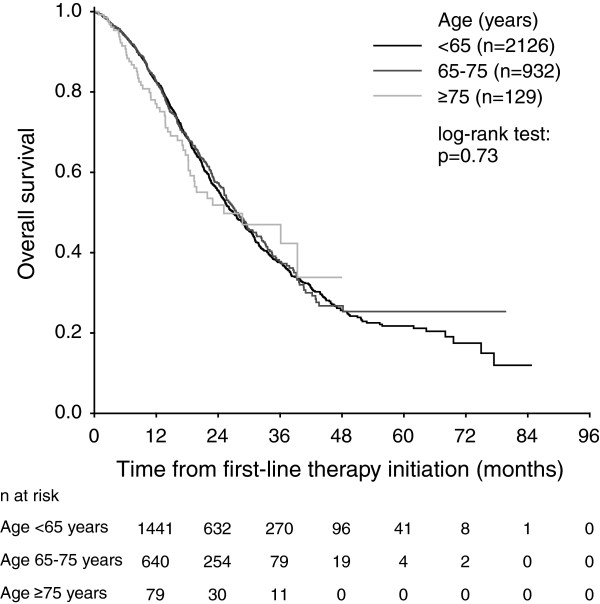
Overall survival of metastatic colorectal cancer patients treated with first-line bevacizumab according to the age categories.

Median PFS was 11.4 months (95% confidence interval [CI] 10.9-11.9 months) for patients aged <65 years, 11.3 months (95% CI 10.5-12.0 months) for patients aged 65 to 75 years, and 11.8 months (95% CI 9.6-14.0 months) for patients ≥75 years (Figure 1). The PFS differences between age categories were not statistically significant (p = 0.94). Median OS was 26.9 months (95% CI 25.3-28.5 months) for patients aged <65 years, 27.5 months (95% CI 25.0-29.9 months) for patients aged 65 to 75 years, and 25.1 months (95% CI 11.3-38.9 months) for patients aged ≥75 years (Figure 2). No statistically significant differences in OS were observed between the age groups (p = 0.73). PFS and OS estimates according to age categories and chemotherapy backbone regimens are presented in Table 2.

**Table 2:** Progression-free survival (PFS) and overall survival (OS) according to age categories and chemotherapy backbone regimens

**Chemotherapy regimen**		**<65 years**	**65-75 years**	**≥75 years**
FOLFOX	n	903	394	51
	Median PFS (95% CI)	11.2 (10.3-12.0)	12.1 (10.9-13.4)	11.8 (8.9-14.7)
	Median OS (95% CI)	25.5 (22.8-28.1)	30.7 (27.6-33.7)	not reached
XELOX	n	753	313	25
	Median PFS (95% CI)	11.5 (10.6-12.5)	11.5 (10.3-12.8)	13.2 (10.9-15.5)
	Median OS (95% CI)	30.0 (27.3-32.7)	27.0 (23.8-30.2)	25.1 (16.6-33.6)
FOLFIRI	n	199	74	5
	Median PFS (95% CI)	12.2 (10.7-13.8)	11.3 (9.0-13.6)	-
	Median OS (95% CI)	25.4 (21.5-29.4)	22.9 (19.9-25.9)	-
XELIRI	n	127	39	1
	Median PFS (95% CI)	14.9 (12.5-17.2)	11.3 (8.4-14.1)	-
	Median OS (95% CI)	29.1 (22.4-35.9)	26.7 (19.9-33.6)	-
Capecitabine	n	39	44	18
	Median PFS (95% CI)	9.4 (6.1-12.7)	10.3 (4.1-16.5)	13.4 (7.9-18.8)
	Median OS (95% CI)	31.1 (27.0-35.2)	17.0 (6.3-27.7)	19.8 (11.8-27.9)
5-FU/LV	n	21	21	23
	Median PFS (95% CI)	10.1 (4.1-16.1)	5.9 (3.3-8.5)	10.5 (6.9-14.0)
	Median OS (95% CI)	22.6 (14.0-31.2)	21.3 (5.4-37.1)	19.4 (10.7-28.2)

In order to adjust for the effect of other potential predictive and prognostic factors that may be associated with age, multivariable Cox model for both PFS and OS was designed (Table 3). Similarly to the univariate analysis, the patient age was not significantly associated with PFS in the multivariable model. When patients <65 years were used as the reference, the hazard ratio (HR) was estimated to be 0.99 (p = 0.88) and 1.01 (p = 0.96) in patients aged 65 to 75 years and patients aged ≥75 years, respectively (Table 3). Variables significantly associated with PFS in the final model included the presence of two and more metastatic sites, synchronous mCRC, and rectal primary.

**Table 3:** Results of the multivariable Cox model for progression-free survival (PFS) and overall survival (OS)

**Model for PFS**	**Risk/baseline category**	**Beta**	**HR**	**95% CI**	**p-value**
Number of metastatic sites	2/1	0.29	1.34	1.22-1.46	<0.001
	3 and more/1	0.53	1.70	1.50-1.94	<0.001
Presence of metastasis at diagnosis	M1/M0	0.12	1.13	1.03-1.23	0.008
Site of primary tumour	Rectum/Colon	0.09	1.10	1.01-1.19	0.04
Age	65-75 years/<65 years	−0.01	0.99	0.91-1.09	0.88
	>75 years/<65 years	0.01	1.01	0.81-1.25	0.96
**Model for OS**	**Risk/baseline category**	**Beta**	**HR**	**95% CI**	**p-value**
Number of metastatic sites	2/1	0.34	1.41	1.25-1.59	<0.001
	3 and more/1	0.66	1.94	1.65-2.27	<0.001
Presence of metastasis at diagnosis	M1/M0	0.23	1.25	1.12-1.41	<0.001
Site of primary tumour	Rectum/Colon	0.12	1.13	1.01-1.26	0.03
Age	65-75 years/<65 years	−0.02	0.98	0.87-1.11	0.73
	>75 years/<65 years	0.17	1.18	0.89-1.56	0.24

*Abbreviations:* HR: hazard ratio; CI: confidence interval.

Similarly, in the multivariable Cox model for OS (Table 3), the patient age was not significantly associated with OS (patients aged 65 to 75 years: HR = 0.98, p = 0.73; patients aged ≥75 years: HR = 1.18, p = 0.24). On the other hand, the number of metastatic sites, the presence of metastatic disease at the diagnosis of CRC, and the site of primary tumour were observed to be the strongest independent predictors of OS. Patients with three and more metastatic sites at the start of bevacizumab therapy were found to have almost two times higher risk of death compared to patients with only one metastatic site, and in patients with two metastatic sites the risk of death was increased by more than 40%. Patients with synchronous metastases had the risk of death increased by 25% compared to patients with metachronous mCRC.

The multivariable Cox models for PFS and OS were also calculated on the subset of patients with available information on performance status (n = 1,821). Similarly to the entire cohort, the patient age was not observed to have significant effect on either PFS or OS (data not shown), whereas the number of metastatic sites and the presence of metastatic disease at diagnosis were confirmed as the strongest prognostic factors with respect to both PFS and OS.

### Safety outcomes

Only bevacizumab-associated toxicity events were reported to the registry. Safety data are summarised in Table 4. As expected, the most common bevacizumab-related adverse events were hypertension and thromboembolic events. Hypertension was reported in 71 patients (3.3%), 34 patients (3.6%), and 10 patients (7.8%) in <65 years, 65 to 75 years, and ≥75 years age group, respectively. Thromboembolic event was reported in 65 (3.1%), 36 (3.9%), and 4 (3.1%) patients aged <65 years, 65 to 75 years, and ≥75 years, respectively. The incidence of both proteinuria and bleeding did not exceed 2.0% in any age group. Gastrointestinal perforation was recorded in 8 patients (5 aged <65 years, 3 aged 65 to 75 years). Severe (i.e. grade ≥ 3) adverse events were rarely observed except for hypertension and thromboembolic events, which were, however, reported in less than 4.0% of patients across all age groups.

**Table 4:** Incidence of bevacizumab-related adverse events

		**All patients**	**<65 years**	**65-75 years**	**≥75 years**
		**(n = 3,187)**	**(n = 2,126)**	**(n = 932)**	**(n = 129)**
		**n (%)**	**n (%)**	**n (%)**	**n (%)**
**New or worsening hypertension**	All	115 (3.6)	71 (3.3)	34 (3.6)	10 (7.8)
	G3-5	51 (1.6)	31 (1.5)	16 (1.7)	4 (3.1)
**Thromboembolic event**	All	105 (3.3)	65 (3.1)	36 (3.9)	4 (3.1)
	G3-5	82 (2.6)	49 (2.3)	30 (3.2)	3 (2.3)
**Proteinuria**	All	59 (1.9)	40 (1.9)	17 (1.8)	2 (1.6)
	G3-5	13 (0.4)	8 (0.4)	5 (0.5)	0 (0)
**Bleeding**	All	40 (1.3)	24 (1.1)	15 (1.6)	1 (0.8)
	G3-5	19 (0.6)	12 (0.6)	7 (0.8)	0 (0)
**Gastrointestinal perforation**	All	8 (0.3)	5 (0.2)	3 (0.3)	0 (0)
	G3-5	4 (0.1)	3 (0.1)	1 (0.1)	0 (0)

## Discussion

The present retrospective observational study using the population-based CORECT registry that included more than 1,000 mCRC patients aged ≥65 years ranks among the largest studies published so far analysing the outcome of treatment with bevacizumab combined with chemotherapy in the elderly patients. Although patients ≥65 years of age represent the majority of patients with mCRC, elderly patients are commonly underrepresented in prospective randomized clinical trials, and, in addition, there are still limited data from observational studies about the efficacy and safety of bevacizumab-containing therapy in this patient population.

With regard to the survival outcomes, the present analysis shows that elderly mCRC patients receiving bevacizumab-containing therapy, both 65 to 75 years and ≥75 years age groups, have PFS and OS similar to those of mCRC patients aged <65 years. These results were confirmed in both the univariate analysis and in the multivariable Cox model adjusted for possible confounding factors. The observation that patient age does not significantly influence PFS and OS of mCRC patients is consistent with previously published reports [11-14]. Thus, patient age should not be considered a limiting factor with respect to bevacizumab-containing therapy in mCRC patients. The most significant factors with respect to both PFS and OS were the number of metastatic sites and the presence of metastases at diagnosis of CRC.

In this study, the median PFS estimates for all age-defined cohorts were higher than the PFS estimates in the BRiTE observational study [15]. Similarly, age-specific median OS estimates in the present analysis were higher compared to the BRiTE study. These differences can be partly explained by an almost 20% higher proportion of patients with synchronous metastases in the BRiTE study. In addition, different distribution of ECOG PS categories could also contribute to the differences in OS. While almost all (97.5%) mCRC patients with recorded performance status information in our cohort had a performance status of 0 or 1, the respective percentage in the BRiTE cohort was lower (92.4%). Moreover, the relatively favourable OS results may also be partly attributed to the effective centralisation of mCRC patients into comprehensive cancer centres (CCCs) which has been implemented in the Czech Republic as of 2006 [11]. The administration of the most expensive cancer drugs including bevacizumab is currently concentrated to only 13 CCCs. In comparison, the median number of patients enrolled per centre was only 8 for the 248 sites in the BRiTE study [8].

Other findings regarding the administration and outcomes of anti-tumour therapy in elderly mCRC patients were consistent with the BRiTE study. As for chemotherapy backbone regimens, the elderly patients received, in general, less aggressive therapy, and both oxaliplatin and irinotecan were administered less often in mCRC patients >65 years of age than in younger patients. In addition, the overall duration of treatment was shorter in the elderly mCRC population compared to younger patients [15]. The tendency to administer less aggressive and shorter therapy can be justified by the fact that the elderly mCRC patients were reported to experience significantly greater hematologic toxicity [17].

Regarding patients ≥75 years of age who were treated with bevacizumab and capecitabine only, the results can be compared with data of the AGITG MAX trial [14] and the AVEX trial [18]. In this analysis, we observed higher median PFS estimate and similar median OS estimate compared with the corresponding estimates published in the two trials (Table 2). However, the difference in PSF should be assessed with caution as only 18 patients were included in the present analysis resulting in high variability of the estimate.

Present results cannot be compared to the Surveillance, Epidemiology, and End Results-Medicare linked database analysis of Meyerhardt et al. [19] that evaluated the effectiveness of first-line bevacizumab-containing therapy in stage IV CRC patients aged >65 years. Firstly, a different time period was evaluated in the U.S. study (mCRC patients diagnosed in 2007 or earlier). Secondly, only patients diagnosed with stage IV CRC (synchronous metastases) were analysed and, thirdly, only patients treated with either oxaliplatin- or irinotecan-based chemotherapy were considered by Meyerhardt et al.

Among the major bevacizumab-related toxicities, new or worsening hypertension, thromboembolic events, and proteinuria have been reported in ≥10% of patients receiving bevacizumab [13,15,20,21]. Moreover, the incidence of hypertension was also found to be age-related [22]. Bleeding, gastrointestinal perforations, and wound healing complications were observed less often [23]. In the present study, the above mentioned adverse events were recorded less frequently than in previously reported (Table 4). Underreporting cannot be obviously excluded in the registry. On the other hand, when considering severe adverse events only (grade ≥ 3); the results of the present study are more consistent with those of other reports. This implies that serious adverse events or events leading to the treatment interruption or modification were more likely to be reported to the database. Despite the lower incidence of adverse events, however, no increase in bevacizumab-related toxicity among the elderly mCRC patients was observed, the only exception being hypertension, which occurred in approximately twice as many patients aged ≥75 years in comparison with patients in <65 years and 65 to 75 years age groups.

The present analysis has several limitations that can be partly attributable to its observational nature. First and foremost, the selection bias cannot be excluded as only medically fit patients with very good performance status might have been treated with bevacizumab-containing therapy among the elderly mCRC patients in contrast to younger patients where indication criteria tend to be less strict. This is suggested by the low proportion of elderly patients in the whole cohort of bevacizumab-treated patients and in turn might have led to more favourable OS estimate in elderly patients. In fact, while patients aged ≥ 65 years represent the majority of mCRC population (64% of newly diagnosed mCRC patients in 2006–2010 according to the Czech National Cancer Registry [2]), in the present analysis only about a third of the patients were aged ≥ 65 years. Secondly, the PFS estimates could have been biased by the fact that neither independent monitoring nor centralized review of radiological response was performed in our study. Last but not least, in comparison with other clinical and observational studies, the adverse events seem to be underreported in the study database. Moreover, only adverse events thought to be linked to bevacizumab were consistently reported in the registry. Although many countries have now implemented some degree of centralisation of treatment and decision-making in the area of targeted cancer therapies, the results presented here may not be fully generalisable to more decentralised health systems. Nevertheless, the 5-year relative survival of patients diagnosed with stage IV colorectal cancer was 11.5%, a figure similar to European and US data [24-26].

## Conclusions

The present large retrospective study confirms that a selected group of elderly mCRC patients fit for chemotherapy combined with targeted therapy may derive similar benefit in terms of improvement in OS and PFS from bevacizumab therapy compared to younger patients. Thus, chronological age should not be considered an exclusion criterion for bevacizumab-containing therapy in mCRC.

## Competing interests

JT, TB, BM, and JF have received speakers’ honoraria from Roche. All other authors state that they have no conflicts of interest.

## Authors’ contributions

LS and TP designed the study, performed the statistical analysis, and wrote the manuscript; LD designed the study and participated in the manuscript writing and in the interpretation of results; ZB and OM participated in the statistical analysis; JT, TB, BM, RV, JP, and JF validated input data as expert oncologists and participated in the interpretation of results and reviewed the manuscript. All authors read and approved the final manuscript.

## Pre-publication history

The pre-publication history for this paper can be accessed here:

http://www.biomedcentral.com/1471-230X/14/53/prepub
